# Bacillus Calmette-Guérin (BCG) vaccine: A global assessment of demand and supply balance

**DOI:** 10.1016/j.vaccine.2017.12.010

**Published:** 2018-01-25

**Authors:** Tania Cernuschi, Stefano Malvolti, Emily Nickels, Martin Friede

**Affiliations:** a World Health Organization, Expanded Programme on Immunization, 20 Avenue Appia, 1211 Geneva, Switzerland; b MMGH Consulting, Kürbergstrasse 1, 8049 Zürich, Switzerland; c Linksbridge SPC, 808 Fifth Ave N, Seattle, WA 98109, United States

**Keywords:** BCG, Tuberculosis, Shortages, Stock-outs, Supply, Demand

## Abstract

Over the past decade, several countries across all regions, income groups and procurement methods have been unable to secure sufficient BCG vaccine supply. While the frequency of stock-outs has remained rather stable, duration increased in 2014–2015 due to manufacturing issues and attracted the attention of national, regional and global immunization stakeholders. This prompted an in-depth analysis of supply and demand dynamics aiming to characterize supply risks. This analysis is unique as it provides a global picture, where previous analyses have focused on a portion of the market procuring through UN entities. Through literature review, supplier interviews, appraisal of shortages, stock-outs and historical procurement data, and through demand forecasting, this analysis shows an important increase in global capacity in 2017: supply is sufficient to meet forecasted BCG vaccine demand and possibly buffer market shocks. Nevertheless, risks remain mainly due to supply concentration and limited investment in production process improvements, as well as inflexibility in demand. Identification of these market risks will allow implementation of risk-mitigating interventions in three areas: (1) enhancing information sharing between major global health actors, countries and suppliers, (2) identifying interests and incentives to expand product registration and investment in the BCG manufacturing process, and (3) working with countries for tighter vaccine management.

## 1 Introduction

Tuberculosis (TB) is one of the major causes of death worldwide, claiming 1.8 million lives in 2015, particularly among communities which already face socioeconomic challenges [1]. Mycobacterium tuberculosis (MTB), the etiological agent of TB, is transmitted via respiratory droplets by patients who are already infected. In 90 percent of infected persons, the bacterium is contained by the host immune response as a latent TB infection (LTBI). Bacillus Calmette-Guérin (BCG) is the only available vaccine to fight the disease, with a duration of protection of at least ten years with some residual vaccine effectiveness up to 20–25 years [2], [3]; however, the vaccine only prevents acute forms of childhood TB, and not reactivation of LTBI (the main source for adult pulmonary disease and transmission of MTB) [1]. WHO recommends universal vaccination with a single birth dose of BCG in settings where TB is highly endemic or where there is high risk of exposure to TB [4].^1^ There is evidence that BCG also prevents leprosy [5], a skin-neurological disease caused by Mycobacterium leprae (200,000 cases in 2016, mainly in Southeast Asia) [6]. BCG vaccine is also effective against other mycobacterial infections, such as Buruli ulcer disease [7].

Over the ninety years since its development, the BCG vaccine has been administered to more than three billion children in the Expanded Programme on Immunization (EPI) across all regions [8]. Although reviews show little evidence that revaccination with BCG affords additional protection, several countries do report implementation of a two-dose schedule [9].^2^

All the BCG vaccines currently in use derive from the original strain of BCG produced by Albert Calmette and Camille Guérin in 1924 at the Pasteur Institute. The original strain was distributed to several countries, leading to generation of the many substrains used today [10]. Currently, the main substrains used for vaccine production are Brazilian (Moreau/Rio de Janeiro), Danish (Copenhagen – 1331), Japanese (Tokyo – 172-1), Russian (Moscow – 368) and Bulgarian (Sofia – SL222). Different strains tend to be used interchangeably, with no conclusive evidence existing to discriminate for efficacy and safety [11]. The choice of the strains used in the different countries is therefore the result of historical use, production, logistics or other factors [12].

Beyond the open questions on efficacy and interchangeability, continued supply availability has been a main challenge with BCG vaccine. The problem has become more acute in recent years: in 2015, UNICEF reported a supply shortfall of 16.5 million doses due to decreased supply capacity. Large middle-income countries (MICs), typically self-procuring, have also experienced issues in accessing supply [13], as have several high-income countries (HICs); in particular, this was a result of the production problems faced by one historical and large supplier (Statens Serum Institut of Denmark – SSI) [14].^3^

**Fig. 1:**
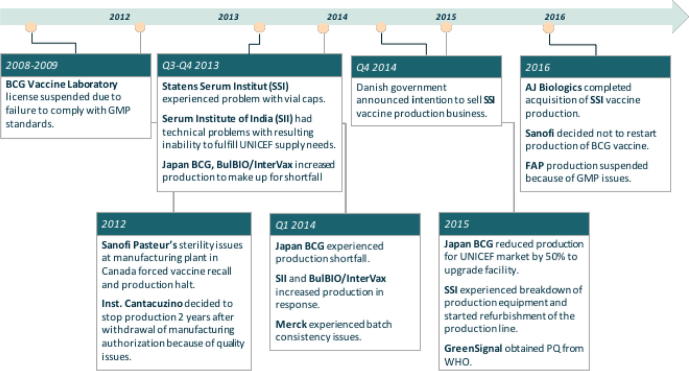
Chronology of key events for BCG vaccine in the period 2008–2016. (Based on unpublished Bill & Melinda Gates Foundation work modified with recent manufacturer, WHO, and UNICEF press releases [13–19].)

Manufacturing problems, in particular GMP issues, and decisions of suppliers to halt their production are not something new to the BCG vaccine market (as illustrated in Fig. 1) [13], [14], [15], [16], [17], [18], [19], [20]. Manufacturing has remained mostly unchanged since the 1920s, with poor characterization and difficulties in maintaining control of the process. The low vaccine price, while affordable for countries, reduced incentives for manufacturers for starting complex and expensive activities for redesign and enhancement of the production process.^4^

This analysis aims to assess the level of risk of BCG vaccine supply in the short- and mid-term and to identify potential areas for intervention. Special attention has been dedicated to maintaining a global perspective: traditional analyses on vaccine availability tend to focus only on segments of the vaccine supply, generally the portion supplied via United Nations (UN) procurement. A global perspective is necessary when assessing vaccine markets, as supply is ultimately allocated on a global basis. In the specific case of BCG vaccine, self-procuring countries account for about 60 percent of total demand.

The analysis investigates BCG vaccine demand and supply dynamics for recent past, present (2017) and near future, with the goal of informing country choices and global policy decisions.

## 2 Methods

The work has been structured in five areas, as described below.

### 2.1 Vaccine shortages

A review of the extent and frequency of vaccine shortages over the past decade was conducted. For this work, vaccine shortages are defined as the inability of countries to meet national needs (population needs plus a required buffer). Nevertheless, in the absence of a precise measure of shortages, the analysis reviews country-reported data on national-level stock-outs^5^ from the WHO/UNICEF Joint Reporting Form (JRF) for the period 2005–2015 [9]. For more recent years (2016 through early 2017), information on shortages has been obtained through consultations with the WHO regional offices.

### 2.2 Global demand

Modelling of global BCG vaccine demand was completed. Annual demand is calculated using the formula below:

[Target Population×Number of Doses×Coverage×Wastage]+Buffer

**Fig. 2:**
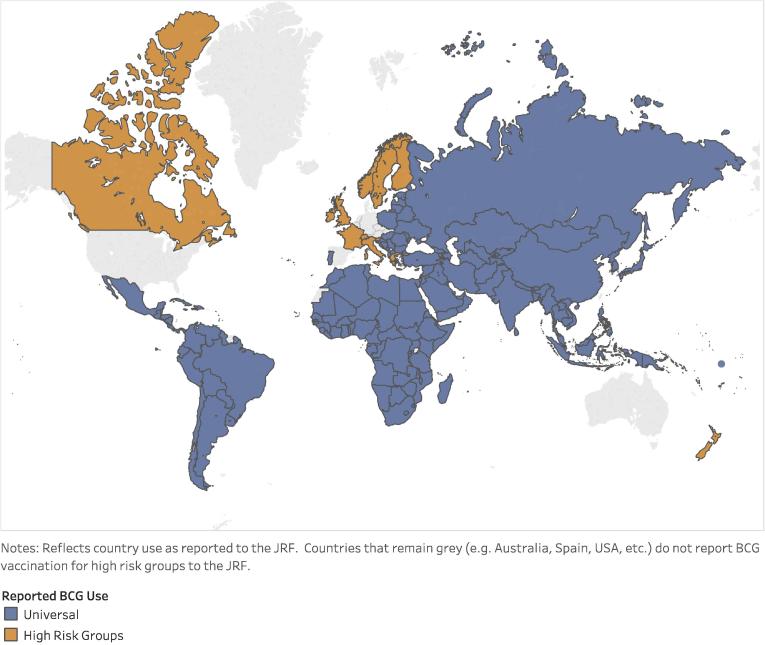
Country-reported BCG use [9].

All 194 WHO Member States report EPI schedules through the WHO/UNICEF JRF on an annual basis [9].^6^ The currently reported schedules for countries reporting universal vaccination (see Fig. 2) were used to identify country-specific target ages as well as number of doses. The UN Population Division (UNPD) population forecast by year of life was used as the country target population for BCG vaccination [21].^7^ The target population was multiplied by the WHO/UNICEF estimated national immunization coverage (WUENIC) [9] and the standard WHO wastage by vial size (in cases of BCG vaccine where the predominant presentations are 10 and 20 dose vials, a factor of 50 percent has been used). The standard buffer stock level (25 percent of the difference in demand between years (positive values only)) was added. The result was a forecast of BCG vaccine demand, per country, per year, for the period 2017–2030 [22].

In addition, data on historical BCG vaccine procurement was available for 146 countries (JRF and UNICEF [23]). This data was compared to the demand forecast for the same 146 countries to inform estimates.

Given the evidence that BCG vaccine can prevent leprosy [24],^8^ we analyzed the EPI schedules for the 22 highest burden countries [9]. In addition, the 17 countries with the next highest case detection rates were also included in our assessment, for a total of 39 countries.^9^ For these countries, we reviewed recommendations for BCG vaccination to estimate potential additional demand.

Finally, to assess the potential impact of migration flows on global demand of BCG vaccine, we looked at the estimates of the number of refugees/migrants arriving in countries/regions with BCG vaccination for high-risk groups; e.g., Canada (in 2015 and 2016, ∼250,000 annually [25]). Vaccination of the entire migrant population with one dose of BCG vaccine was then assumed, given lack of comprehensive data on BCG vaccination practices for these populations. In connection with the recent migrant crisis in the European Union, we estimated the influx of migrants (in 2016, estimates range from 362,000 to 1.2 million [26], [27]) and assumed one dose for each, independent of country policy.

### 2.3 Global supply

A list of manufacturers with available supply of BCG vaccine (and product characteristics) has been compiled with the help of PAHO Revolving Fund, International Federation of Pharmaceutical Manufacturers & Associations (IFPMA) and Developing Countries Vaccine Manufacturers Network (DCVMN), as well as through a review of published literature [12], [28], [29], [30], [31], [32], [33], UNICEF Supply Division market updates [13],^10^ and internal reports from Bill & Melinda Gates Foundation (BMGF) and Clinton Health Access Initiative (CHAI).

All manufacturers were contacted to obtain information on available products and their characteristics (presentation, shelf-life, route of administration, strain, disease and age indication), as well as information on countries of registration and manufacturing capacity. Nine interviews took place, including all five WHO prequalified (PQ’d) producers.^11^

This process allowed for building of three supply estimates: the historical view of 2015 supply, the current available supply for 2017 and a future short-term projection for 2019. The available information indicates that no new major vaccine is to be expected into the market before 2020 and therefore new product availability is unlikely to impact the current BCG vaccine market.

### 2.4 Product registration

For each of the identified products, information has been collected from manufacturers on their registration status in different countries. This data was combined with information on source of procurement (JRF) and on country ability to accept the collaborative procedure between WHO and NRAs for the assessment and accelerated national registration of WHO PQ’d pharmaceutical products and vaccines [34]. This information was used to estimate countries’ ability to readily access alternative products in case of supply failure.^12^

### 2.5 Global demand-supply balance

Finally, demand and supply estimates were compared to validate historical dynamics and to identify ongoing and future challenges for access.

Results were discussed as part of the proceedings of the WHO Strategic Advisory Group of Experts Working Group on BCG vaccine. They are presented in this paper at the aggregate level to ensure compliance with confidentiality agreements and anti-trust regulations.^13^

## 3 Results

### 3.1 Vaccine shortages

The analysis confirms that several BCG stock-outs were reported through the JRF across all regions and income groups: an average of 43 out of 194 countries per year experienced stock-outs from 2005 to 2015.^14^ The average duration of stock-outs for those countries was 1.4 months for the period 2005–2013. In 2014 and 2015, the average duration increased to 2.6 and 2.8 months, respectively. Stratification of the data provides useful insights:

• The African region has been most affected – annually, an average of 41 percent of countries in the region experienced stock-outs versus 12 to 22 percent of countries in other regions.
• Low-income countries (LICs) and lower-middle-income countries (LMICs) were also more affected, with an average of 41 and 34 percent of countries in these income groups experiencing stock-outs yearly, versus 17 and 4 percent for upper-middle-income countries (UMICs) and high-income countries (HICs), respectively.^15^
• Different procurement methods seem not to modify risk of stock-out.

As mentioned, we cannot attribute each stock-out to a shortage of vaccine supply. Consultation with WHO regional offices confirmed that at times, availability of financing, national or external, was an issue. Local procurement shortcomings and ineffective vaccine management were also quoted among reasons for stock-outs. Nevertheless, supply issues are also an important factor behind countries’ inability to meet supply needs.

Countries and international agencies leveraged various coping strategies to deal with the unavailability of supply:

• UNICEF worked with manufacturers (with and without PQ’d products) to access supply not yet allocated. Vaccine distribution was prioritized using a process established by WHO and UNICEF [35]. WHO worked with manufacturers on prequalification expectations and a new product was PQ’d in 2015.^16^
• In countries, different strategies were implemented to ensure uninterrupted supply, from reducing shipment size to tighter stock management and changes in vaccination practices to reduce wastage. Alternative suppliers were also sought and used where possible: temporary import licenses from manufacturers were arranged for products not registered. Despite these efforts, over the years, some countries had to suspend vaccination for a few months until vaccine became available.

### 3.2 Global demand

**Fig. 3:**
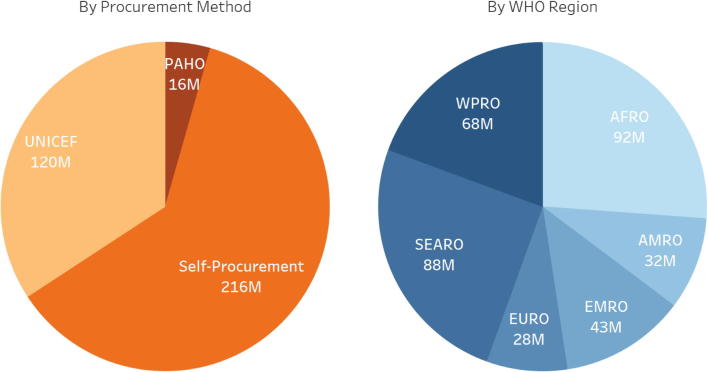
2017 Adjusted Global Demand (352 million doses) by Procurement Method and WHO Region. (For reference, the birth cohorts, by procurement method, are: Self-procuring – 78 M, UNICEF – 47 M, PAHO – 5 M [21].)

The result of the global demand forecast was an annual average demand of 227 million doses.^17^ However, comparison with historical procurement data suggested this was an underestimate. While on average about 290 million doses of BCG vaccine were procured in the period 2006–2015 for the 146 countries with data available in JRF,^18^ modeled demand for these same 146 countries accounted for an average of approximately 190 million doses per year. The amount of JRF-reported procured doses is 1.5 times greater than forecasted demand, with the greatest differences recorded in the self-procuring LMICs. Accounting for larger than expected past purchases, the annual global demand forecast of 229 million doses was increased by 1.5 times to an estimated 350 million (Fig. 3).

Since all 39 countries with high leprosy burden recommend universal BCG vaccination for prevention of TB [9], no additional BCG demand for prevention of leprosy was included in the global demand forecast.

Finally, the additional demand due to migration was estimated to be negligible (less than 1 percent of forecasted global demand).

**Fig. 4:**
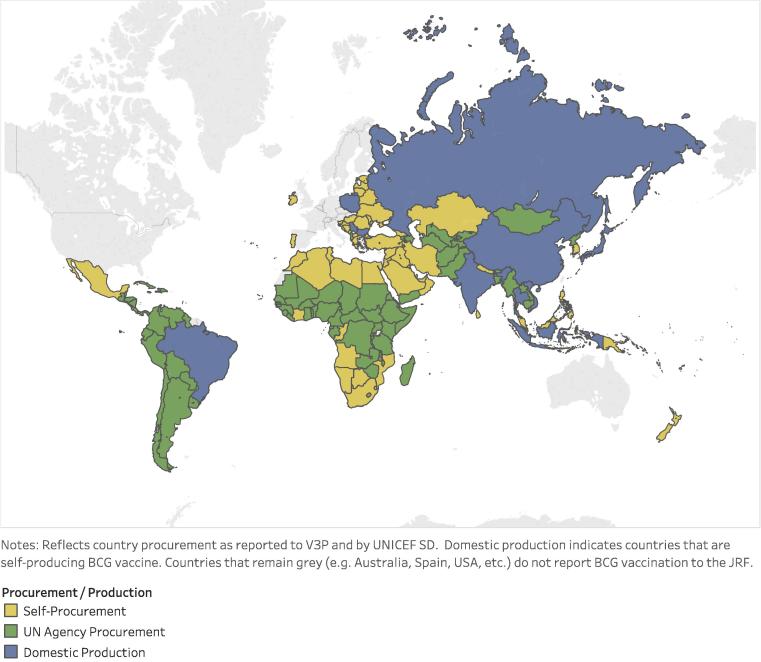
Reported country BCG procurement and production. (For reference, the 2017 birth cohort of self-producing countries is 55.3 million [21].)

Review of demand analysis shows that the largest share (60 percent) of demand for BCG vaccine is from countries self-procuring supply. UNICEF Supply Division procures for many countries, representing about 35 percent of demand, while the PAHO Revolving Fund accounts for the remaining five percent (see Fig. 4). Over 60 percent of forecasted BCG vaccine demand for 2017 is for 20-dose vials: UNICEF procures 20-dose vials only, PAHO procures 10-dose vials, China produces/procures 5-dose vials, and self-procurement is split between 10- and 20-dose vials [9], [36], [37]. UN agency procurement is exclusively of WHO PQ’d products; self-procuring countries procure both PQ’d and non PQ’d products.

### 3.3 Global supply

Twenty-two manufacturers (as listed in Table 1) were identified as having manufacturing capacity available for BCG vaccine for prevention of tuberculosis, including two manufacturers belonging to the same corporate group.^19^ It is important to notice that all but two currently active manufacturers are in countries with National Regulatory Authorities (NRAs) deemed functional by WHO.^20^

**Table 1:** Current BCG vaccine manufacturers (March 2017).^a^

Manufacturer	Country	Strain	Supplying beyond domestic market	PQ Status	Releasing NRA functionality	Vial size	Route of administration	Supplying in 2017
ANLIS	Argentina	Pasteur 1173 P2 strain	N	N	N	10ds/20ds	Unknown	Y
Fundação Ataulpho de Paiva	Brazil	Moreau, Rio strain	Y	N	Y	10ds	Unknown	N
BulBio-NCIPD^b^	Bulgaria	Bulgarian substrain (Sofia) SL222	Y	Y	Y	10ds/20ds	Intradermal	Y
China National Biotec Group (Shanghai)	China	Chinese substrain Shanghai D2PB302 (derived from Danish strain 823)	Y	N	Y	5ds	Intradermal	Y
China National Biotec Group (Chengdu)	China	Chinese substrain Shanghai D2PB302 (derived from Danish strain 823)	Y	N	Y	5ds	Intradermal	Y
Shaanxi Pharmaceutical Holding Group	China	Chinese substrain Shanghai D2PB302 (derived from Danish strain 823)	N	N	Y	20ds	Unknown	Y
AJ Biologics	Denmark	Danish 1331 strain	Y	Y	Y	10ds/20ds	Intradermal	N
Serum Institute of India	India	Russian (Moscow) – 368	Y	Y	Y	10ds/20ds	Intradermal	Y
Green Signal Biopharma	India	Danish 1331 strain	Y	Y	Y	20ds	Intradermal	Y
BCG Vaccine Laboratory, Chennai	India	Danish 1331 strain, Madras Working Seed Lot (MWSL)	Y	N	Y	10ds/20ds	Intradermal	N
Taj Pharma Ltd	India	Russian (Moscow) – 368	N	N	Y	20ds	Unknown	Y
BioFarma	Indonesia	Pasteur 1173P strain	Y	N	Y	5ds/10ds	Intradermal	Y
Pasteur Institute of Iran	Iran	Pasteur 1173P2 strain	N	N	Y	20ds	Intradermal	Y
Japan BCG Laboratory (JBL)	Japan	Tokyo 172-1 strain	Y	Y	Y	10ds/20ds	Intradermal/percutaneous (multipuncture device)	Y
Biomed Lublin	Poland	Moreau strain	Y	N	Y	10ds	Intracutaneous	Y
Microgen	Russia	Russian (Moscow) – 368	Y	N	Y	10ds/20ds	Intradermal	Y
Inst. Of Virology, Vaccines and Sera Torlak	Serbia	Pasteur 1173P2 strain	Y	N	N	10ds/20ds	Intradermal	Y
NIIDV	Taiwan	Tokyo 172 strain	N	N	Y	20ds	Intradermal	Y
Queen Saovabha Mem. Inst (Thai Red Cross)	Thailand	Japanese strain	N	N	Y	10ds	Intradermal	Y
Inst. Pasteur Tunis	Tunisia	Pasteur 1173P2 strain	N	N	Y	20ds	Intradermal	Y
Merck & Co (former Organon)	United States	TICE strain	N	N	Y	1ds	Percutaneous (multipuncture device)	Y
IVAC – Institute of Vaccines and Medical Biologicals	Vietnam	Pasteur 1173P2 strain	N	N	Y	20ds	Unknown	Y

a Compiled with the help of PAHO Revolving Fund, International Federation of Pharmaceutical Manufacturers & Associations (IFPMA) and Developing Countries Vaccine Manufacturers Network (DCVMN), as well as through a review of published literature [12,28–33], UNICEF Supply Division market updates [13], and internal reports from the Bill & Melinda Gates Foundation (BMGF) and Clinton Health Access Initiative (CHAI).

b BulBio vaccine is also distributed by InterVax.

For 2017, the global capacity is estimated to be approximately 500 million doses *—* a 30 percent increase compared to 2015 and well above expected demand. Capacity is split into two groups:

• Four producers with PQ’d products (Serum Institute of India, GreenSignal, Japan BCG Laboratory and InterVax/BB-NCIPD) that can reach 169 countries where their products are registered or UN procurement is accepted; and
• Fifteen producers with non-PQ’d products that can serve 52 countries where their product is registered.

Consultations with manufacturers suggest a potential for further increase of 35 percent in the next two years, allowing supply to reach 700 million doses, thanks to the return of the three manufacturers currently not supplying the market (Table 1) and flexibility in capacity of some of the manufacturers already supplying the market.

### 3.4 Product registration

Results show that more than half of the world’s countries accept WHO PQ’d product either via direct UN procurement or through the collaborative procedure between WHO and NRAs for regulatory approval. Consequently, these countries have the potential for quick access to a large supplier base (all four PQ’d products and, in some cases, also non-PQ’d products with local licenses) which provides a safety net in case of supply issues.

About 20 countries require local licensure (are not accepting PQ’d products) and have two or three products licensed. These countries can switch between the in-country licensed products in case of supply issues.

Importantly, over 40 countries require local licensure and have only one product registered. In these countries, discontinuation or reduction in supply from the manufacturer of their only licensed product can lead to supply issues. This high-risk group can be further stratified by looking at (a) whether countries have local BCG vaccine production *—* and are therefore better informed about and in control of potential issues *—* and (b) whether countries without local production have BCG vaccine in their EPI schedule *—* thus requiring large supplies. This latter group is composed of about 20 countries.

A second group with high risk is a set of 12 countries that, at the time of the analysis, appear to have no product registered. All are HICs whose preferred supplier has withdrawn from the market. None of these countries use BCG vaccine in their EPI schedule and could procure with a temporary import license to address needs. While some risk remains, there are certainly more resources and established processes to access information and more quickly address supply needs than in lower-income countries.

## 4 Discussion

### 4.1 Historical procurement and estimated demand

Results of demand analysis show 2016 country-reported procurement is 1.5 times greater than 2017 forecasted demand. Several hypotheses have been formulated on the potential drivers; one of these is that actual wastage may be higher than 50 percent based on recent information on country immunization sessions.^21^ Other possible explanations: countries may be holding stocks larger than the recommended buffer, reported country data may also be including uses beyond prevention of tuberculosis (e.g., oncology).

### 4.2 Global demand-supply balance

In 2017, available supply of BCG vaccine (∼500 M doses) is forecasted to be 1.5 times greater than forecasted demand (∼350 M doses) and countries are not reporting supply access issues.

Despite the manufacturer exits of the past 20 years, BCG vaccine has an unusually large number of suppliers, if we consider that most of the currently produced vaccines count on low (single-digit) numbers of producers. This large supplier base is the result of a simple production process that has allowed several countries to maintain production for self-sufficiency. Also, given the low economic attractiveness of the BCG vaccine market, small manufacturers have been able to survive longer and no consolidation efforts from larger producers are noted.

Unfortunately, this large supply base has been very unstable: as a result of the outdated production processes and limited investments, manufacturing issues, often quality and GMP related, have forced manufacturers to frequently suspend production or exit the market.

Given the instability of the BCG vaccine manufacturing process, this extra supply is sufficiently reassuring and shows important progress relative to the bleaker supply/demand balance of recent years.

### 4.3 Risk identification

Despite some progress, the BCG vaccine market is not risk free; this analysis identified several issues of concern around access to BCG vaccine supply.

First, two suppliers continue to represent more than half of global vaccine supply and, importantly, 75 percent of supply PQ’d by WHO. The complete loss of a major supplier will not automatically lead to a supply/demand imbalance, but it will certainly create a situation of constrained supply, requiring tight and careful management. In such a circumstance, since those large suppliers are critical both to self-procuring countries and countries procuring through UNICEF, it will be necessary to carefully manage information sharing and supply allocations across these two groups.

Second, results show a large dependency on one NRA: the two largest BCG vaccine suppliers are currently released by the same NRA (India). While WHO currently deems this NRA functional, this situation should not be overlooked. The return to market of AJ Biologics in the near future could reduce this risk.

Third, countries with only one product registered that do not accept the collaborative procedure between WHO and NRAs for PQ’d products present an issue; these tend to be middle- and upper-income countries. For these countries, a production issue by the only manufacturing source could easily lead to a shortage. Similarly, the higher-income countries with one or no product registered that also do not recommend use of BCG may face challenges in obtaining larger BCG supply to vaccinate migrants from high TB-burden countries. Countries for which concern is higher are those where the BCG vaccine is in the EPI schedule and thus have the largest demand *—* in particular, those countries that import products and do not have much visibility on possible production issues *—* as well as large countries that have less flexibility in coping with shortages through small shipment sizes and stock management.

These identified threats to sustainable access to BCG vaccine supply are important, considering the historical record of BCG vaccine manufacturing issues. Despite some recent small investments in manufacturing aimed at addressing continued GMP issues, it is not evident that production issues will be less likely in the future. Similarly, given the low economic attractiveness of the BCG vaccine market, the risk of further manufacturer exit remains, particularly in light of the apparent abundance of supply.

Finally, while a systematic review of published trials did not find differences in protection against TB between different strains of BCG [38], there is currently no consensus on this issue and further studies are warranted [39], [40], [41]. Results from such studies, as well as evolution of the BCG vaccine pipeline, would certainly influence supply and demand dynamics. A close and continuous monitoring of those studies is warranted to ensure that policies and procurement practices allow sufficient time for supply adjustments.

### 4.4 Opportunities for action

The identified risks also represent valuable opportunities. As consultations with manufacturers, immunization agencies and countries were carried out, it became apparent that global information collection and sharing could help to address and sometimes prevent some issues, such as countries lacking information on the potential capacity to address imminent shortages, or foreseeing the possible impact of supply constraints on a particular group of countries.

A few key investments can also be explored based on identified risks: (i) offering financial/other incentives for strengthening production processes, thus enhancing the overall stability of supply and (ii) creating a ‘BCG vaccine registration fund’ encouraging manufacturers to register products in more locations despite regulatory burden – in the context of longer term efforts to harmonize registration requirements among countries.

One final consideration: the analysis shows that historical demand seems to be much larger than what would be forecasted based on available information on target population, coverage and wastage rates. While this deserves further study – particularly on actual wastage rate – it is possible that some countries could reduce stock levels and improve forecasting and procurement to reduce demand to what is strictly necessary. This could also enhance use of scarce national resources for immunization.

Activities could possibly target specific regions (such the African region) or income groups (LICs and LMICs) which have suffered the most from access issues for this vaccine.

### 4.5 Limitations of study

Some limitations to this analysis should be highlighted. Firstly, while stock-outs remain the most useful information currently available to measure shortages, they can be caused by a range of issues (e.g., supply shortages, but also financing delays, inaccurate forecasting and inefficient stock management or procurement). Furthermore, supply shortages do not always lead to stock-outs.^22^ Secondly, supply data are fully relying on manufacturers’ input and have not been validated through other independent sources nor facility assessments. Thirdly, product licensure information has not been validated with each country, with a risk of information being incomplete. Finally, country-reported data through the WHO/UNICEF JRF was heavily leveraged for the analysis: data quality varies, particularly in relation to historical procurement, but remains the only available data at present.

## 5 Conclusion

The BCG vaccine market experienced several national stock-outs over the past decade across all regions, income groups and procurement methods. While the frequency has remained rather stable, duration of stock-outs increased in 2014–2015 due to manufacturing issues and attracted the attention of national, regional and global immunization stakeholders. This analysis shows an important increase in global capacity in 2017, with sufficient supply to meet forecasted BCG vaccine demand and possibly buffer market shocks. However, risks remain that are related to supply concentration and limited demand flexibility in a market characterized by high instability and low investment. Through a collaboration among immunization partners, various avenues can be explored to address identified risks, particularly by enhancing information sharing, working with countries to improve vaccine management, and working with manufacturers and donors to understand options to tackle production risks and registration issues.

## Footnotes

1 WHO is currently reviewing its position on BCG and will publish an updated position paper in early 2018.

2 Reporting countries: Bulgaria, Kazakhstan, Russia, Seychelles, Tajikistan, Turkmenistan, Ukraine.

3 SSI completed divestment of its BCG vaccine business to AJ Biologics in 2016 after having experienced repeated production and GMP issues.

4 Analysis of BCG vaccine price by income, region, manufacturer, volume and procurement method can be generated from the V3P website at http://apps.who.int/immunization/vaccineprice (last accessed 07 April 2017).

5 Stock-out of a vaccine for at least one month indicates that “safety stocks have been depleted and vaccine availability for the national immunization programme could be compromised” [42].

6 WHO/UNICEF JRF data includes country-reported EPI schedules and estimated coverage (WUENIC) and can be viewed or downloaded here: http://www.who.int/immunization/monitoring_surveillance/data/en/.

7 The total birth cohort for 153 countries with BCG in EPI is 131 million [21].

8 Of note: as of October 2017, WHO recommends universal birth dose vaccination with BCG in high burden leprosy settings regardless of the TB incidence (SAGE report: http://apps.who.int/iris/bitstream/10665/259533/1/WER9248.pdf?ua=1).

9 Data on leprosy case detection rates provided by the WHO Global Leprosy Programme (GLP).

10 All UNICEF Supply Division market updates are available here: https://www.unicef.org/supply/index_54214.html.

11 Capacity of manufacturers that did not respond (all focused on supplying local markets) has been estimated based on the corresponding local market size. Based on the input from manufacturers, doses allocated for oncological use have been excluded.

12 This data is unpublished and available through WHO Global Polio Eradication Initiative.

13 The SAGE WG on BCG vaccine was established in October 2016. For more information, please consult WHO’s website at: http://www.who.int/immunization/policy/sage/sage_wg_bcg_oct2016/en/ (last accessed 07 April 2017).

14 These also include producing countries.

15 According to the 2016 World Bank income classification.

16 WHO determines the acceptability, in principle, of vaccines from different sources for supply to United Nations agencies. The detailed procedure can be found here: http://www.who.int/immunization_standards/vaccine_quality/pq_system/en/ (last accessed 04 April 2017).

17 The 2017 modelled demand for the 153 countries with BCG vaccine in the EPI schedule is 229 million doses. The target population is 128 M and includes live births and booster doses in seven countries. The seven countries with booster doses have a total birth cohort of ∼3 M.

18 Historical procurement data was submitted for 48 countries through the JRF.

19 Chengdu Institute for Biological Products and Shanghai Institute for Biological Products both belong to China National Biotech Group (CNBG).

20 WHO has developed a benchmarking system using a benchmarking tool with established standard criteria for evaluating NRAs in the area of vaccine regulation, and has identified six key regulatory functions that NRAs need to perform within a regulatory system defined by law. http://www.who.int/immunization_standards/national_regulatory_authorities/role/en/.

21 Also, a recent analysis of the use of 20-dose vials of BCG vaccine in Guinea-Bissau indicates that only 13 to 15 doses can be withdrawn from this very common presentation, leading to a larger need than usually forecasted [43].

22 A country could have sufficient in-country stock to continue vaccinating during the shortage period.
